# The Absence of Toll-Like Receptor 4 Mildly Affects the Structure and Function in the Adult Mouse Retina

**DOI:** 10.3389/fncel.2019.00059

**Published:** 2019-02-27

**Authors:** Agustina Noailles, Oksana Kutsyr, Victoria Maneu, Isabel Ortuño-Lizarán, Laura Campello, Emilio de Juan, Violeta Gómez-Vicente, Nicolás Cuenca, Pedro Lax

**Affiliations:** ^1^Department of Physiology, Genetics and Microbiology, University of Alicante, Alicante, Spain; ^2^Department of Optics, Pharmacology and Anatomy, University of Alicante, Alicante, Spain; ^3^Institute Ramón Margalef, University of Alicante, Alicante, Spain

**Keywords:** TLR4 knockout mice, electroretinography, visual acuity, immunohistochemistry, transmission electron microscopy

## Abstract

The innate immune Toll-like receptor (TLR) family plays essential roles in cell proliferation, survival and function of the central nervous system. However, the way in which TLRs contribute to the development and maintenance of proper retinal structure and function remains uncertain. In this work, we assess the effect of genetic TLR4 deletion on the morphology and function of the retina in mice. Visual acuity and retinal responsiveness were evaluated in TLR4 knockout and wild type C57BL/6J control mice by means of an optomotor test and electroretinography, respectively, from P20 to P360. Retinal structure was also analyzed in both strains using confocal and electron microscopy. ERG data showed impaired retinal responsiveness in TLR4 KO mice, in comparison to wild type animals. The amplitudes of the scotopic a-waves were less pronounced in TLR4-deficient mice than in wild-type animals from P30 to P360, and TLR4 KO mice presented scotopic b-wave amplitudes smaller than those of age-matched control mice at all ages studied (P20 to P360). Visual acuity was also relatively poorer in TLR4 KO as compared to C57BL/6J mice from P20 to P360, with significant differences at P30 and P60. Immunohistochemical analysis of retinal vertical sections showed no differences between TLR4 KO and C57BL/6J mice, in terms of either photoreceptor number or photoreceptor structure. Horizontal cells also demonstrated no morphological differences between TLR4 KO and wild-type mice. However, TLR4 KO mice exhibited a lower density of bipolar cells (15% less at P30) and thus fewer bipolar cell dendrites than the wild type control mouse, even though both confocal and electron microscopy images showed no morphologic abnormalities in the synaptic contacts between the photoreceptors and second order neurons. Microglial cell density was significantly lower (26% less at P30) in TLR4 KO mice as compared to wild-type control mice. These results suggest that TLR4 deletion causes functional alterations in terms of visual response and acuity, probably through the loss of bipolar cells and microglia, but this receptor is not essential for the processing of visual information in the retina.

## Introduction

Toll-like receptors (TLRs) are type I transmembrane proteins that mediate innate immune responses triggered by pathogen-associated (PAMP) and damage-associated molecular pattern (DAMP) molecules (Oda and Kitano, 2006; West et al., 2006; Kawai and Akira, 2007). Ten human and thirteen mammalian TLRs have been characterized to date (Akira et al., 2006; McGuire et al., 2006; Kumar, 2018), and are primarily expressed in tissues associated with the immune function, although they are also expressed by other non-immune cells including glia and neurons (Hanke and Kielian, 2011). It is now evident that, far from their primary well-known function as mediators of the immune response, TLRs play additional relevant roles in the progression of different pathological conditions, such as non-infectious neuronal injuries and neurodegenerative disorders (Carty and Bowie, 2011; Hanke and Kielian, 2011; Okun et al., 2011; Feldman et al., 2015). Interestingly, recent findings show that TLRs also appear to be involved in many physiological cellular processes, such as the development of neuronal circuits during embryogenesis, progenitor cell proliferation and differentiation, CNS plasticity throughout the entire life or drug-reward behavior (Rolls et al., 2007; Shechter et al., 2008; Okun et al., 2011; Barak et al., 2014; Kashima and Grueter, 2017; Grasselli et al., 2018). So far, numerous endogenous TLR ligands have been identified that could play a role in the regulation of CNS physiological processes and in the development and progression of neurodegenerative disorders (Trotta et al., 2014).

Toll-like receptors also influence neuronal survival. This effect depends on the receptor subtype and localization, and can be directly exerted in the brain and the retina through TLR activation in neurons (Ma et al., 2006, 2007; Yi et al., 2012), progenitor cells (Shechter et al., 2008) and glia (Trudler et al., 2010). The activation of TLR4 in retinal microglia and Müller cells can directly affect the viability of photoreceptors (Yi et al., 2012). In addition, several studies have shown that TLR4 modulates neuronal survival under conditions such as CNS non-infectious diseases and injuries, neuroinflammation and degenerative disorders like multiple sclerosis, Alzheimer’s and Parkinson’s diseases, and amyotrophic lateral sclerosis (Mollen et al., 2006; Tang A.H. et al., 2007; Tang S.C. et al., 2007; Walter et al., 2007; Shi et al., 2017; Zhang et al., 2018). TLR4 might also affect the survival of newborn neurons. Intraperitoneal injection of the natural TLR4 ligand lipopolysaccharide (LPS) during the neonatal period has been shown to decrease the survival of dividing astrocytes and neurons in the hippocampus of mice (Jarlestedt et al., 2013). But, interestingly, activation of TLR4 before the occurrence of a harmful stimulus might induce preconditioning and has a neuroprotective effect, which opens the door to new therapeutic approaches using TLRs agonists (Marsh et al., 2009; Yi et al., 2012).

In the mammalian retina, TLR expression has been demonstrated in astrocytes (Jiang et al., 2009), microglia (Maneu et al., 2011), Müller cells (Kumar and Shamsuddin, 2012; Lin et al., 2013; Sauter et al., 2018), photoreceptors (Yi et al., 2012), bipolar, amacrine and ganglion cells (Qi et al., 2014; Sauter et al., 2018), retinal pigmented epithelium (Kumar et al., 2004; Ebihara et al., 2007) and vascular endothelial cells (Stewart et al., 2015). TLRs mediate the immunological responses in the eye (Luo et al., 2010; Kleinman and Ambati, 2012; Kohno et al., 2013; Klettner et al., 2014; Liu and Steinle, 2017; Coburn et al., 2018). But, to date, there is a lack of information regarding their influence on retinal development and neuronal plasticity. A previous work from Shechter and collaborators revealed that TLR4 acts as a negative regulator of the proliferation and neuronal differentiation of retinal progenitor cells in the ciliary margin and *ora serrata* (Shechter et al., 2008) in the early postnatal period. However, no studies have yet been conducted to determine whether TLR4 deletion affects the morphology and function of the mouse retina during growth, maturation and aging.

Given the role of TLRs in neurogenesis, proliferation of progenitor cells and neuronal differentiation, the main objective of this work was to determine whether TLR4 deletion had any effects on the structure and/or function of the mouse postnatal retina, and if so, whether these potential effects would vary throughout growth, maturation and aging. Increasing our knowledge of the involvement of TLRs in physiological and pathological conditions may provide new therapeutic options for both infectious and non-infectious diseases.

## Materials and Methods

### Animals

TLR4 KO mice, kindly provided by Dr. M.L. Gil and Dr. D. Gozalbo (Universitat de València, Spain), were employed in this study (Hoshino et al., 1999). Age-matched wild-type C57BL/6J mice (Harlan Laboratories, Barcelona, Spain) represented the control animals. All animals were housed in cages under controlled photoperiod (12 h light/12 h dark), temperature (23 ± 1°C) and humidity (55 to 60%). Food and water were provided *ad libitum*. All the procedures were performed according to Project License UA-2013-07-22, which was approved by the Ethics Committee for Animal Experimentation from the University of Alicante. All animals were treated according to current regulations on the use of laboratory animals (NIH, ARVO and European Directive 2010/63/UE) in an effort to minimize animal suffering and the number of animals used.

### Electroretinography

We recorded scotopic and photopic ERG responses at ages P20 and 1, 2, and 12 months, essentially as has been previously described (Lax et al., 2011). After a period of adaptation to overnight darkness, procedures were carried out to prepare the animals for bilateral ERG recording under dim red light. An intraperitoneal injection of ketamine (100 mg/kg) and xylazine (4 mg/kg) was administered as anesthesia, and the animals were kept on a heating pad at 38°C. A topical application of 1% tropicamide (Alcon Cusí, Barcelona, Spain) was administered to dilate their pupils. A drop of Viscotears 0.2% polyacrylic acid carbomer (Novartis, Barcelona, Spain) was placed on the cornea to facilitate electrical contact with the recording electrodes and to prevent dehydration. DTL fiber electrodes were used, consisting of an X-Static silver-coated nylon conductive strand and supplied by Sauquoit Industries (Scranton, PA, United States). The reference electrode was a 25-gauge platinum needle placed between the eyes under the scalp. A gold electrode placed in the animal’s mouth served as ground. Anesthetized animals were put in a Faraday cage and all experiments were conducted in total darkness. Scotopic flash-induced ERG responses to light stimuli produced by a Ganzfeld stimulator were recorded in both eyes. Light stimuli were administered at 11 different increasing luminances (ranging from -5.2 to 0 log cd⋅s/m^2^) for 10 ms each. The mean of three to ten consecutive recordings was calculated for each light administration. An interval of 10 s was left between flashes for dim flashes (-5.2 to -1.4 log cd⋅s/m^2^) and as much as 20 s for higher luminances (-0.8 to 0 log cd⋅s/m^2^). After a 20-min period of light adaptation at 10 cd/m^2^, photopic responses were obtained using the same stimuli as for scotopic conditions. ERG signals were first amplified and then band-pass filtered (1–1000 Hz, without notch filtering) by means of a DAM50 data acquisition board (World Precision Instruments, Aston, United Kingdom). A PowerLab system (AD Instruments, Oxfordshire, United Kingdom) was used for the administration of stimuli and data acquisition (4 kHz). Recordings were saved to a computer file and were subsequently analyzed off-line. To visualize oscillatory potentials, the signal recorded was filtered between 100 and 1000 Hz. The a-wave amplitude measurement was taken from the baseline 10 ms after the onset of the light stimulus, i.e., prior to the intrusion of the b-wave. The b-wave amplitude measurement was taken from the trough of the a-wave to the peak of the b-wave. For oscillatory potentials the maximum peak-to-trough amplitude was considered.

### Optomotor Test

The spatial frequency threshold was assessed for awake, freely moving TLR4 KO and C57BL/6J mice at ages P20 and 1, 2, and 12 months. The Argos system (Instead, Elche, Spain) was used to observe and score optomotor responses to horizontally drifting, vertically oriented gratings. The spatial frequency threshold for the behavior was considered to be the maximum spatial frequency at maximum contrast that was still capable of inducing smooth head tracking movements. For this test, a mouse was positioned on a platform in the center of a chamber whose sides were four computer monitors. Sinusoidal gratings were projected on all monitors as a virtual cylinder centered on the head and rotating in both horizontal directions. An overhead infrared video camera was used by a trained observer to record the mouse and score smooth head turns in response to the rotating gratings. These tracking responses were observed to be robust at middle spatial frequencies and diminished until they disappeared at the threshold.

### Retinal Histology

Retinal histological studies were carried out at P20, P30 and 12 months of age following well-established procedures (Noailles et al., 2014). Briefly, the animals were administered a lethal dose of pentobarbital in the mornings. After marking the dorsal margin of the limbus by means of a suture, the eyes were enucleated and fixed in 4% (w/v) paraformaldehyde during 1 h at room temperature. They were then washed in 0.1 M phosphate buffer pH 7.4 (PB) and sequentially cryoprotected in 15, 20, and 30% (w/v) sucrose. The cornea, lens and vitreous body were excised, and the eyecups were then embedded in Tissue-Tek OCT (Sakura Finetek, Zoeterwouden, Netherlands) and frozen in liquid N_2_. Sections with a thickness of 16 μm were obtained at -25°C in a cryostat, mounted on slides (Superfrost Plus; Menzel GmbH and Co. KG, Braunschweig, Germany) and stored at -20°C. Before subsequent use, slides were thawed, washed 3 times with PB and incubated for 1 h with blocking solution [10% (v/v) donkey serum and 0.5% (v/v) triton X-100 in PB]. For single or double immunostaining, sections were incubated overnight at room temperature with combinations of antibodies at different dilutions in PB with 0.5% Triton X-100. The primary antibodies used (see Table 1) have already been characterized in other works. For an objective comparison, TLR4 KO and C57BL/6J retinas were processed in parallel. Donkey anti-mouse or anti-rabbit IgG conjugated to Alexa Fluor 488 or 555 (1:100, Molecular Probes, Eugene, OR, United States) were used as secondary antibodies. In some cases, the nuclear marker TO-PRO 3 iodide (Molecular Probes) was added at a dilution of 1:1000. Images were taken under a Leica (Leica Microsystems, Wetzlar, Germany) TCS SP2 confocal laser-scanning microscope using a spatial resolution of 1024 × 1024. The pinhole was set at 1 airy unit and z-stacks were made of 15 pictures (1.5 μm steps). All stacks were taken under 40× magnification, with an acquisition rate of 16 frames per second. Adobe Photoshop 10 software (Adobe Systems Inc., San Jose, CA, United States) was used to process the final images from C57BL/6J and TLR4 KO groups in parallel.

**Table 1:** Primary antibodies employed in this work.

Molecular marker	Antibody (reference)	Source	Dilution
Bassoon	Mouse monoclonal (Fernandez-Sanchez et al., 2012)	Enzo Life Sciences (ADI-VAM- PS003)	1:1000
Calbindin D-28K	Rabbit polyclonal (Lax et al., 2014)	Swant (CB-38a)	1:500
Cone arrestin	Rabbit polyclonal (Noailles et al., 2018)	Millipore (AB15282)	1:500
Ionized calcium-binding adapter molecule 1 (Iba1)	Rabbit polyclonal (Noailles et al., 2014)	Wako Chemicals (019-19741)	1:1000
Major histocompatibility complex class II RT1B (clone OX-6) (MHCII)	Mouse monoclonal (Noailles et al., 2016)	AbD Serotec (MCA46R)	1:200
Protein kinase C, α isoform (α-PKC)	Rabbit polyclonal (Pinilla et al., 2016)	Santa Cruz Biotechnology (sc-10800)	1:100
RBPMS	Rabbit polyclonal (Gomez-Vicente et al., 2015)	Millipore (ABN1362)	1:500
Rhodopsin	Mouse monoclonal (Pinilla et al., 2016)	Millipore (MAB5356)	1:500

### Transmission Electron Microscopy

Twelve-month-old C57BL/6J and TLR4 KO mice were perfused with 4% paraformaldehyde and 2% glutaraldehyde in 0.1 M PB. Eyes were enucleated and immersed in the same solution for 2 h. After 2 rinses in 0.1 M PB, eyes were dissected, excising the cornea, iris, lens and vitreous body, and cut into four pieces. Each piece was postfixed in 1% osmium tetroxide (OsO_4_) in 0.1 M PB for 1 h. After being gradually dehydrated in a sequence of ethanol and acetone solutions, the pieces were embedded in EPON 812 overnight. EPON blocks containing the retinal pieces were polymerized at 60°C overnight. Ultrathin sections were obtained with an ultramicrotome (Leica Ultracut R, Leica Microsystems), and lead citrate and uranyl acetate were used for contrast. Transmission electron microscopy images were obtained in a 120 kV JEOL JEM-1400 microscope (JEOL GmbH, München, Germany).

### Morphometric Analysis

Measurement of the thickness of the retinal layers, ribbon density and retinal cell counting were performed using the NIH ImageJ software developed by Wayne Rasband (National Institutes of Health, Bethesda, MD, United States^1^). Differences in immunofluorescence signals were analyzed by obtaining the corresponding grayscale intensity (range 0–256) profile plots, using the NIH ImageJ software, and quantifying the area (in pixels) under the intensity profile. In all sections analyzed, quantification was performed close to the optic nerve. At least four animals per experimental group were analyzed.

For the quantification of bipolar cell dendrites, four square zones of 4047 pixel square were analyzed per image in the OPL. For ganglion cell counting, in each flat mounted retina twelve equidistant regions of 0.144 mm^2^ in the temporal-nasal and superior-inferior axes. Six regions were arranged on the superior-inferior axis and six fields were disposed on the temporal-nasal axis; representative peripheral, medial and central areas of the superior, inferior, temporal and nasal quadrants of each retina were evaluated.

### Statistical Analysis

A two-way ANOVA was applied to assess the effects of genotype (TLR4 KO vs. C57BL/6J) and experimental stage (P20 and 1, 2, and 12 months old), as well as any interactions between them. *Post hoc* pairwise comparisons using Bonferroni’s test were carried out when a 0.05 level of significance was obtained. Normal distributions and homogeneity of variance were seen for the categories of the previously defined variables. Values of *P* < 0.05 were considered to be statistically significant. Data were plotted as the average ± standard error of the mean (SEM). All statistical analyses were conducted using SPSS 22.0 software (Statistical Package for Social Sciences, Chicago, IL, United States).

## Results

### TLR4 Deletion Decreases Retinal Responsiveness

**FIGURE 1:**
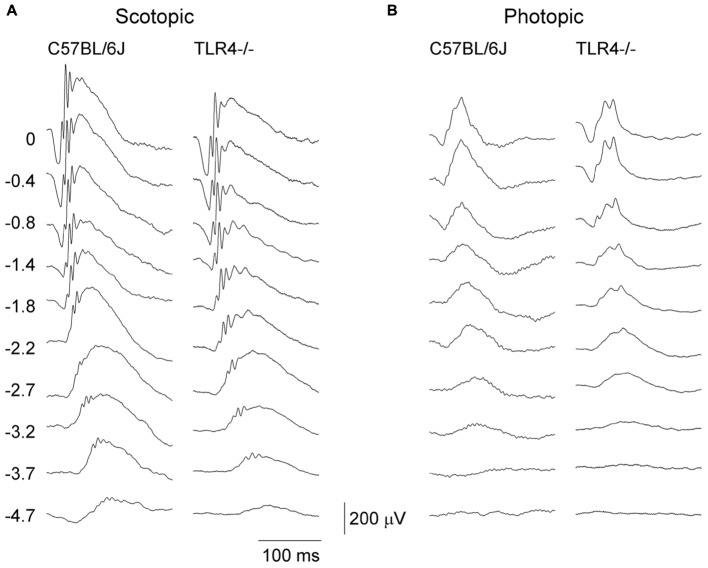
Electroretinographic responses in control and TLR4-deficient mice. Representative scotopic **(A)** and photopic **(B)** ERG traces obtained from control (C57BL/6J) and TLR4 KO mice at P30. Units on the left indicate the luminance of the flashes in log cd⋅s/m^2^. Note that scotopic ERG responsiveness was lower in TLR4 KO mice, as compared to the control mice.

To assess the effect of TLR4 deletion on the functioning of the mouse retina, scotopic and photopic flash-induced ERG responses were recorded in both TLR4 KO and wild type animals at P30 (Figure 1), an age when the retina is fully developed. Under scotopic conditions, ERG responsiveness was lower in TLR4 KO mice than in the wild type. The maximum amplitudes observed for scotopic a- and b-waves in TLR4 KO animals were 91 and 87% of the values obtained for wild type animals (ANOVA, Bonferroni’s test; *P* < 0.05 (scotopic a-waves); *P* < 0.001 (scotopic b-waves); *n* = 14 for TLR4 KO mice and *n* = 21 for wild type animals). No significant differences were observed in the a- and b-wave latencies. Under photopic conditions, no significant differences were found between both groups, with maximum amplitudes of photopic a- and b-waves in TLR4 KO animals being 100 and 102% of the values obtained for wild type animals.

**FIGURE 2:**
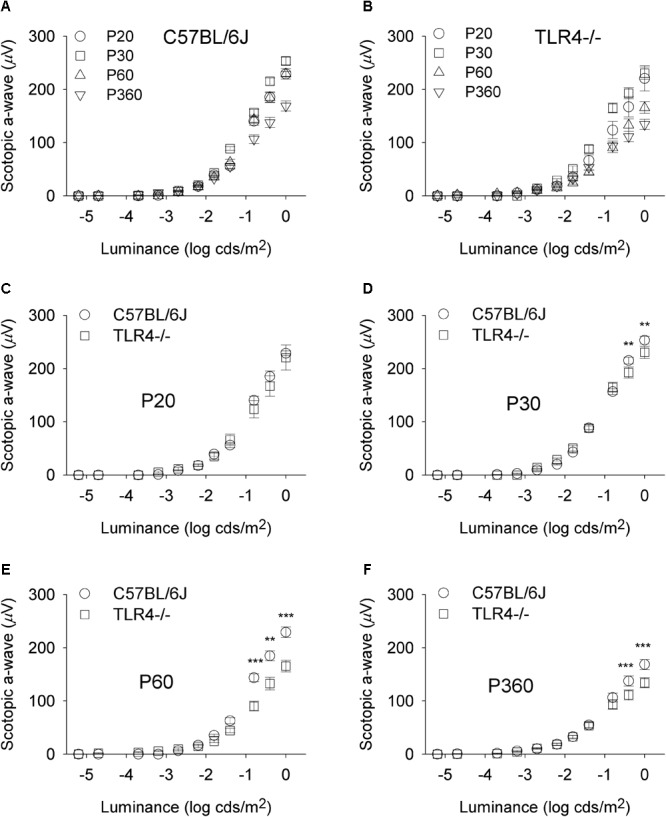
Scotopic a-wave luminance-response curves in control and TLR4-deficient mice. **(A,B)** Response curves for mixed scotopic a-waves obtained from control (**A**, C57BL/6J) and TLR4 KO (**B**, TLR4-/-) mice at different ages, as indicated. **(C–F)** Paired comparisons between C57BL/6J and TLR4-/- mice responses for each light stimulus at P20 **(C)**, P30 **(D)**, P60 **(E)**, and P360 **(F)**. The comparison showed significant differences between the two groups tested at P30, P60, and P360 (ANOVA, Bonferroni’s test; *n* = 6 to *n* = 21 in each group). ^∗^*P* < 0.05, ^∗∗^*P* < 0.01, ^∗∗∗^*P* < 0.001. Error bars represent the SEM.

**FIGURE 3:**
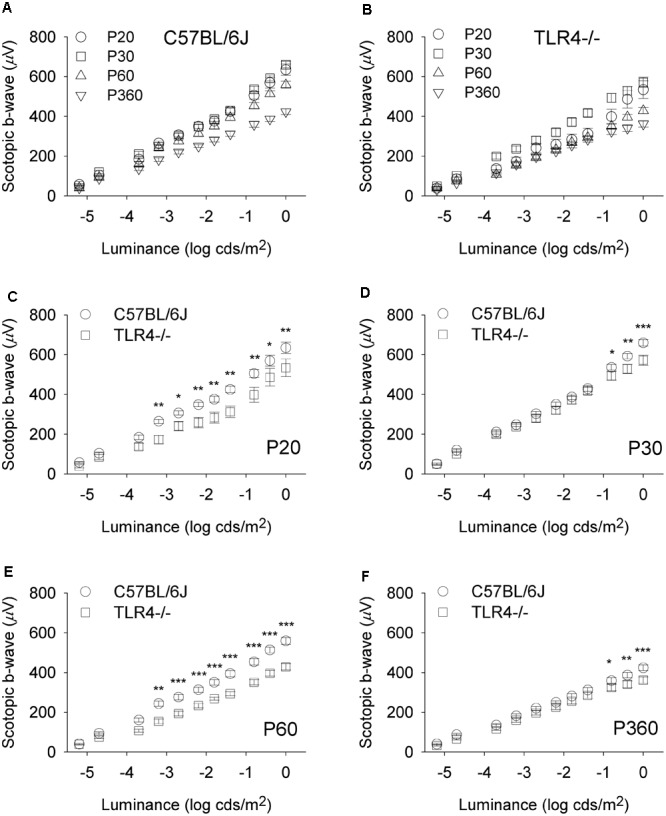
Scotopic b-wave luminance-response curves in control and TLR4-deficient mice. **(A,B)** Response curves for mixed scotopic b-waves obtained from control (**A**, C57BL/6J) and TLR4 KO (**B**, TLR4–/–) mice at different ages, as indicated. **(C–F)** Paired comparisons between C57BL/6J and TLR4–/– mice responses for each light stimulus at P20 **(C)**, P30 **(D)**, P60 **(E)**, and P360 **(F)**. Significant differences are evident between the two groups tested at all conditions (ANOVA, Bonferroni’s test; *n* = 6 to *n* = 21 in each group). ^∗^*P* < 0.05, ^∗∗^*P* < 0.01, ^∗∗∗^*P* < 0.001. Error bars represent the SEM.

**FIGURE 4:**
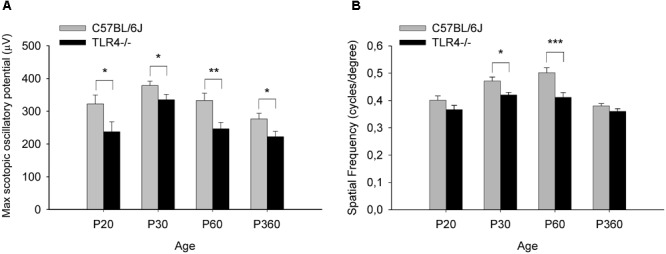
Oscillatory potentials amplitude and visual acuity in control and TLR4-deficient mice. **(A)** Amplitude of maximum oscillatory potentials from control and TLR4 KO mice (ANOVA, Bonferroni’s test; *n* = 6 to *n* = 21 in each group). **(B)** Spatial frequency threshold (in cycles per degree) in the optomotor test at different ages in control (C57BL/6J, gray) and TLR4-deficient (TLR4–/–, black) mice. Visual acuity was slightly lower in TLR4 KO mice, with a significant difference observed between the two groups at P30 and P60 (ANOVA, Bonferroni’s test; *n* = 4 to *n* = 16 in each group). ^∗^*P* < 0.05, ^∗∗^*P* < 0.01, ^∗∗∗^*P* < 0.001. Error bars represent the SEM.

To assess whether the detrimental effect of TLR4 deletion on retinal responsiveness was exacerbated with aging, scotopic flash-induced ERG responses were recorded at different ages (P20 and 1, 2, and 12 months) in both TLR4 KO and wild type animals. Figure 2, 3 show that ERG responsiveness progressively decreased from P20 to 12 months in both control animals (Figure 2A, 3A) and TLR4 KO mice (Figure 2B, 3B). However, the maximum a-wave amplitudes observed in TLR4 KO mice under scotopic conditions were lower than in control animals at all ages tested (Figure 2C–F), with a decrease of 3.6, 9.0, 27.7, and 17.2% at P20 and 1, 2, and 12 months, respectively, which were significantly different at 1, 2, and 12 months of age (ANOVA, Bonferroni’s test, *P* < 0.05 to *P* < 0.001, as indicated in Figure 2C–F; *n* = 6 to *n* = 21 in each group). Also, for scotopic b-waves, the maximum amplitudes recorded were lower in TLR4 KO mice at all ages tested (Figure 3C–F) (decrease of 15.9, 13.4, 23.4, and 14.7% at the corresponding ages), reaching significance at all ages tested (ANOVA, Bonferroni’s test, *P* < 0.05 to *P* < 0.001, as indicated in Figure 3C–F; *n* = 6 to *n* = 21 in each group). Oscillatory potentials in the scotopic ERG from TLR4 KO mice also showed a significantly lower amplitude, compared with control animals, at all ages tested (Figure 4A).

Visual acuity also changed with age in wild type and TLR4 KO animals (P20 and 1, 2, and 12 months), with the mice reaching maximum acuity at 2 and 1 months, respectively (Figure 4B). However, visual acuity values were slightly lower in TLR4 KO mice than in control animals from P20 to 12 months of age, with the differences being statistically significant at 1 and 2 months of age (10.8 and 17.9% less acuity, respectively; ANOVA, Bonferroni’s test, *P* < 0.05 and *P* < 0.001, respectively; Figure 4; *n* = 4 to *n* = 16 in each group). Altogether, these results demonstrate that TLR4 deletion decreases retinal responsiveness in mice.

### TLR4 Deficiency Has No Effect on the Number and Morphology of Photoreceptor Cells

**FIGURE 5:**
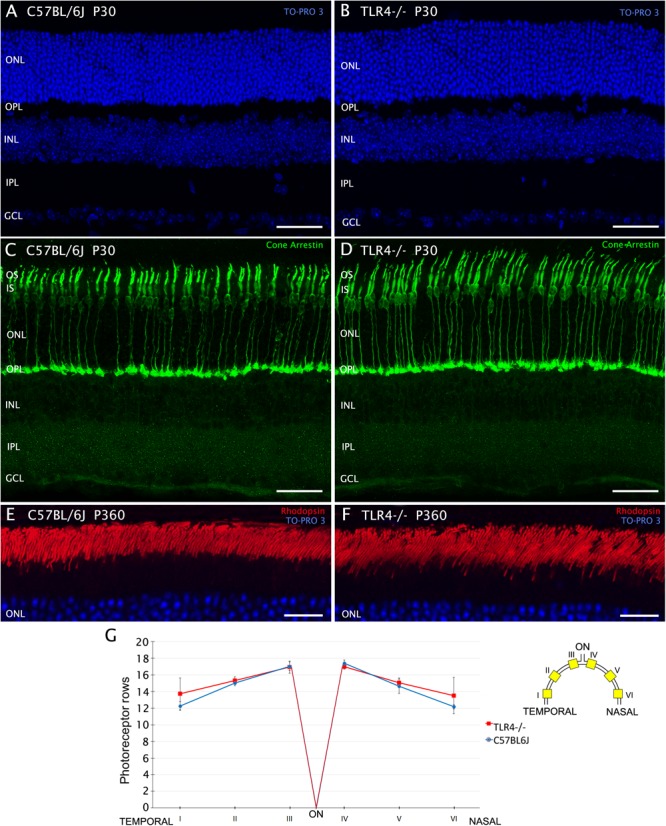
Number and morphology of photoreceptors in control and TLR4-deficient mice. **(A–F)** Vertical sections from control (**A,C,E**; C57BL/6J) and TLR4-deficient (**B,D,F**; TLR4–/–) mice retinas stained with the nuclear marker TO-PRO 3 (blue) to visualize all cell nuclei **(A,B,E,F)**, and labeled for cone arrestin (green) to visualize cone photoreceptors **(C,D)** or for rhodopsin (red) to evidence rod outer segments **(E,F)**. **(G)** Quantitation of photoreceptor rows in the ONL in both C57BL/6J and TLR4–/– retinas (*n* = 5 in both cases). The scheme to the right of the panel shows the position of each representative region analyzed in the retina. Error bars represent the SEM. OS, outer segment; IS, inner segment; ONL, outer nuclear layer; OPL, outer plexiform layer; INL, inner nuclear layer; IPL, inner plexiform layer; GCL, ganglion cell layer; ON, optic nerve. Scale bar: 40 μm.

Given the decrease in retinal functionality detected in TLR4 KO mice, we have investigated whether morphological alterations could be behind this abnormal response. Different immunohistochemical analyses were performed on wild type and TLR4 KO animals. We first determined the average number of photoreceptor rows found in the outer nuclear layer (ONL) of each retina, employing the nuclear dye TO-PRO 3 iodide (Figure 5A,B). Because ONL thickness varies throughout the retina, we examined the effects of TLR4 deletion in several retinal areas, ranging from the temporal to the nasal regions. We found that the average number of photoreceptor rows was similar in control and TLR4 KO animals at P30 (14.8 ± 0.1 and 15.3 ± 0.4, respectively, *n* = 5 in both cases; Figure 5G). Similar results were found at P20 (not shown). To evaluate photoreceptor morphology, we labeled the cones using antibodies against cone arrestin (Figure 5C,D) and observed no morphological alterations in TLR4 KO mice as compared to the wild type. Immunofluorescence images of rhodopsin did not show differences between TLR4 KO and control mouse retinas (Figure 5E,F).

### Absence of TLR4 Reduces the Density of Bipolar Cells and Bipolar Cell Dendrites

**FIGURE 6:**
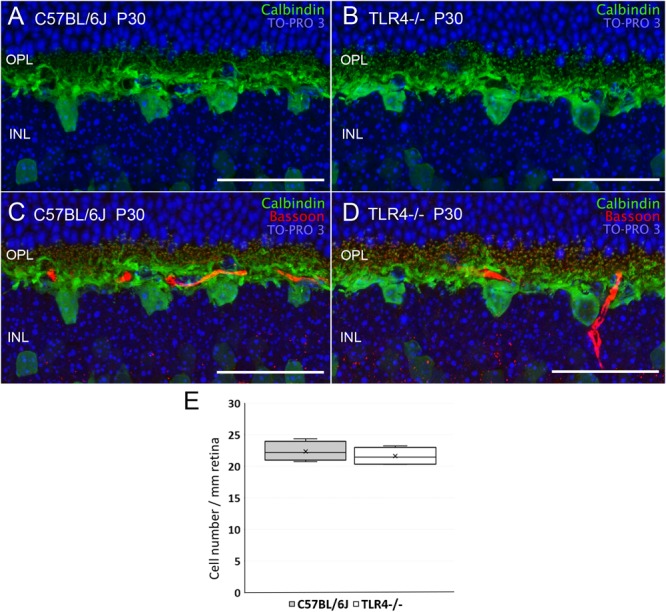
Horizontal cells morphology and quantitation in control and TLR4-deficient mice. **(A–D)** Vertical sections from P30 control (**A,C**; C57BL/6J) and TLR4-deficient (**B,D**; TLR4–/–) mice retinas labeled with antibodies against calbindin (horizontal cells, green) and Bassoon (synaptic ribbons, red). The nuclear marker TO-PRO 3 (blue) was used to visualize all cell nuclei. **(E)** Quantitation of horizontal cells per mm of retinal section in control (gray) and TLR4 KO (white) retinas at P30 (*n* = 5 in both cases). OPL, outer plexiform layer; INL, inner nuclear layer. Scale bar: 40 μm.

Despite the fact that the absence of TLR4 affected neither the number of photoreceptors nor their morphology, retinal responsiveness was impaired, so we wondered whether neurons postsynaptic to photoreceptors, such as bipolar and horizontal cells, could be affected by the lack of TLR4. Horizontal cell bodies are found in the outermost part of the inner nuclear layer (INL). They establish connections with both rod and cone photoreceptors. The only horizontal cells found in the murine retina belong to a subtype that can be labeled using antibodies against calbindin. Calbindin immunostaining of TLR4 KO and wild type retinas showed no differences between the experimental groups (Figure 6A,B) as regards the morphology and density of horizontal cell dendrites. Interactions between horizontal cell dendrites and photoreceptor synaptic ribbons also showed no differences between control and TLR4-deficient mice (Figure 6C,D, respectively). Quantification of the horizontal cell number at P30 showed no significant differences between TLR4 KO and wild type retinas (ANOVA, Bonferroni’s test; Figure 6E, *n* = 5 in both cases).

**FIGURE 7:**
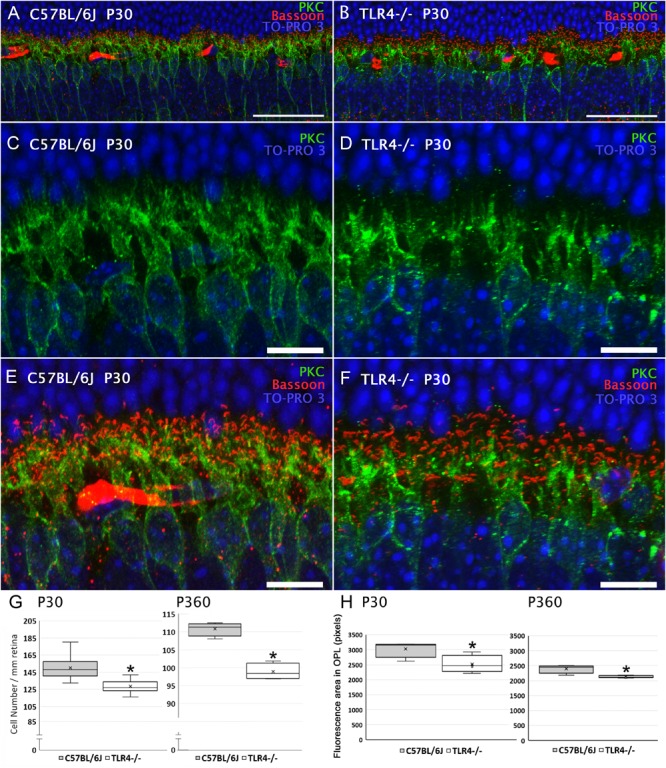
Bipolar cells morphology and quantitation in control and TLR4-deficient mice. **(A,B)** Vertical sections from P30 control (**A**; C57BL/6J) and TLR4-deficient (**B**; TLR4–/–) mice retinas labeled with antibodies against PKC (rod bipolar cells, green) and Bassoon (synaptic ribbons, red). The nuclear marker TO-PRO 3 (blue) was employed to visualize all cell nuclei. **(C–F)** Magnification of **(A,B)** showing the labeling of rod bipolar cells **(C,D)** or the double labeling of rod bipolar cells and synaptic ribbons **(E,F)**. Note that both positive cell bodies and dendrites were more numerous in control animals than in TLR4-deficient mice. **(G)** Quantitation of bipolar cells per mm of retinal section in control (gray) and TLR4 KO (white) retinas at 1 (P30) and 12 months (P360) of age. **(H)** Quantitation of bipolar cell dendrites in the OPL in control (gray) and TLR4 KO (white) retinas. Data are represented as mean values of fluorescence. ^∗^*P* < 0.05; ANOVA, Bonferroni’s test, *n* = 4 to *n* = 12 in each group. Error bars represent the SEM. Scale bar: 40 μm **(A,B)**, 10 μm **(C–F)**.

We next explored alterations in the number and/or morphology of bipolar cells. Rod bipolar cells were identified using an antibody raised against the α isoform of protein kinase C (PKC). In the mouse retina, dendritic terminals of ON-rod bipolar cells make connections with rod spherules by means of a massive dendritic arbor in the outer plexiform layer (OPL) (Figure 7A–F), and their axons extend into the inner plexiform layer (IPL), where each ends in a bulbous axon terminal in the S5 stratum. As shown in Figure 7G, the density of ON-rod bipolar cell bodies in the INL was significantly lower in the retinas of P30 TLR4 KO mice as compared to their age-matched controls (85% of the value obtained for wild type animals; ANOVA, Bonferroni’s test, *P* < 0.05, *n* = 11 for C57BL/6J mice and *n* = 12 for TLR4 KO mice). A similar decay in the number of rod bipolar cells was found in the retinas of P20 TLR4 KO animals (71% of the value obtained for age-matched wild type animals; ANOVA, Bonferroni’s test, *P* < 0.05, *n* = 4 in both cases; data not shown). The analysis of the retinas of 12-month-old animals also revealed a lower number of bipolar cells in TLR4-/- mice as compared to that observed in age-matched wild type animals (89% of the value obtained for wild type animals; ANOVA, Bonferroni’s test, *P* < 0.05, Figure 7G, *n* = 4 in both cases). The lower number of rod bipolar cells in TLR4 KO animals resulted in a lower density of bipolar cell dendrites when compared to wild type animals, with a mean fluorescence value of 83.09% in TLR4 KO animals with respect to that observed in control animals at P30, and 88.75% at the age of 12 months (ANOVA, Bonferroni’s test, *P* < 0.05, Figure 7H, *n* = 4 in both cases).

No significant differences were found in ganglion cell numbers. We found a mean value number of 2149 ± 194 ganglion cells/mm^2^ in C57BL/6J mice and 2044 ± 115 ganglion cells/mm^2^ in TLR4 KO mice.

### TLR4 Ablation Does Not Affect Photoreceptor Synaptic Complexes

Given the significant reduction in the number of rod bipolar cells in TLR4 KO mice, we hypothesized that synaptic connectivity between photoreceptors and second order neurons could be impaired in these animals. To explore this possibility, we used an antibody against Bassoon, a protein component of the synaptic ribbons presents in both the rod spherules and cone pedicles of the OPL. As shown in Figure 6, 7, the typical Bassoon-immunoreactive spots, with the characteristic horseshoe shape that is typical of rod spherules, were observed in a similar proportion in both control and TLR4-deficient mice (Figure 6C, 7E for C57BL/6J mice and Figure 6D, 7F for TLR4 KO mice). Quantification of the photoreceptor synaptic ribbons showed no significant differences between TLR4-/- mice and wild type animals at both ages tested, P30 and P360 (in pixels of fluorescence area in the OPL: 13996 ± 1902 vs. 14578 ± 1040 at P30 and 13165 ± 1633 vs. 13784 ± 1274 at P360).

**FIGURE 8:**
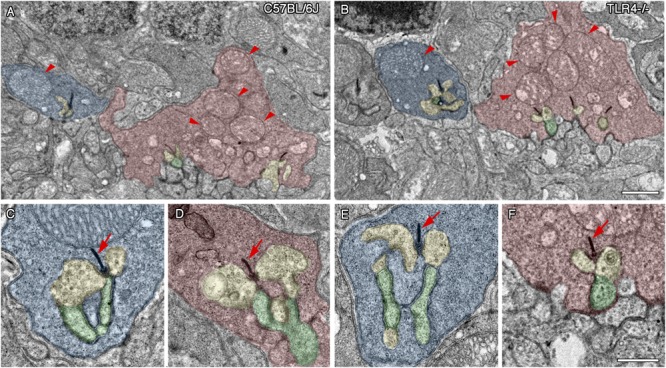
Ultrastructure of photoreceptor ribbon synapses in control and TLR4-deficient mice. Transmission electron microscopy images from 12 months old control (**A,C,D**; C57BL/6J) and TLR4-deficient (**B,E,F**; TLR4–/–) mice retinas. **(A,B)** Representative regions of the OPL of a control **(A)** and TLR4-deficient **(B)** mouse showing normal rod spherules (blue) and cone pedicles (red). Note the typical large mitochondrion in rod spherules and the multiple mitochondria in the cone pedicles (arrowheads). **(C–F)** Higher magnification representative triad synaptic complexes in spherules **(C,E)** and pedicles **(D,F)**. The ribbon synapse and the arciform density are electron-dense structures (arrows) surrounded by two horizontal cell elements (yellow) and a bipolar cell process (Schafer et al.), forming the typical triad structure. No major differences were observed in terms of synaptic complex structure between controls and TLR4 KO mice. Scale bar: 1 μm **(A,B)**, 500 nm **(C–F)**.

With the purpose of analyzing in greater detail whether photoreceptor synaptic complexes are altered by TLR4 ablation, transmission electron microscopy (TEM) images were used to visualize rod spherules and cone pedicles, as well as their synaptic connections to bipolar and horizontal cells in the OPL of 12 months old TLR4 KO and wild type animals. The typical triads, made up by the lateral elements of horizontal cell processes and the central elements of bipolar cells profiles, presented a normal morphology in the rod spherules and cone pedicles of wild type and TLR4 KO animals (Figure 8A,B, respectively). Rod spherules and cone pedicles showed normal electron-dense synaptic ribbons and arciform structures (Figure 8C–F, arrows) in the two experimental groups analyzed. Also, the typical large mitochondrion in rod spherules and the multiple mitochondria in the cone pedicles were found (Figure 8A,B, arrowheads). Together, these findings indicate that synaptic contacts between photoreceptors and second order neurons presented the typical ultrastructural features of a normal retina in TLR4 mice.

### Absence of TLR4 Reduces the Density of Retinal Microglia

**FIGURE 9:**
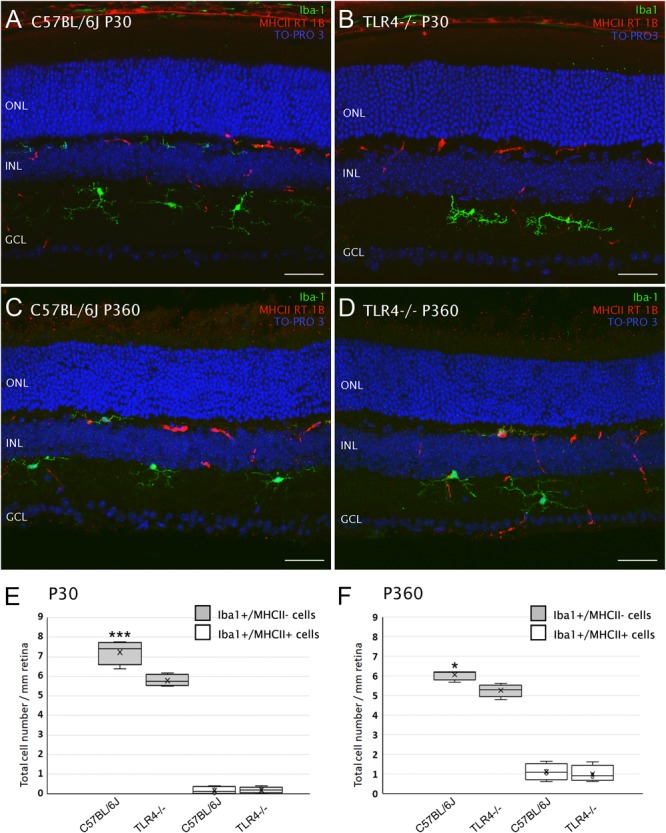
Microglial cells in control and TLR4-deficient mice. **(A–D)** Vertical retinal sections from control (**A,C**; C57BL/6J) and TLR4-deficient **(B,D)** mice at P30 **(A,B)** and P360 **(C,D)** stained for Iba1 (green) and MHCII RT 1B (red). The nuclear marker TO-PRO 3 (blue) was employed to visualize all cell nuclei. **(E,F)** Quantification of cells stained with one or both markers (Iba1/MHCII) per mm of retinal section at P30 **(E)** and P360 **(F)**. ^∗^*P* < 0.05, ^∗∗∗^*P* < 0.001; ANOVA, Bonferroni’s test, *n* = 4 in all cases. Error bars represent the SEM. ONL, outer nuclear layer; INL, inner nuclear layer; GCL, ganglion cell layer. Scale bar: 40 μm.

Bearing in mind that TLR4 plays a role in the innate immune response in the brain, and that microglia serve as the CNS resident immune cells, which includes the retina, we also explored whether TLR4 deletion affected microglial cell numbers in the mouse retina. To that end, vertical retinal sections of 1- and 12-month-old mice were immunostained with an antibody against the ionized calcium-binding adaptor molecule 1 (Iba1). Microglia and macrophages have been reported to express specifically and ubiquitously this calcium-binding protein (Ito et al., 1998; Noailles et al., 2014). As can be seen in Figure 9, microglial cells were found in the inner and outer plexiform cell layers of both wild type and TLR4 KO mice (Figure 9A–D). Iba1-positive cells displayed a very small soma, little perinuclear cytoplasm, and very many fine, branched processes with numerous projections, i.e., the usual morphological features of resting microglia. The appearance of Iba-1 positive cells in immunolabeled retinal sections of TLR4 KO mice was similar to that observed in C57BL/6J control mice. The mean density of Iba1-positive cells, however, was found to be significantly lower in TLR4 KO mice than in their age-matched controls at P30 and P360 (72 and 87% of the value obtained for wild type animals; ANOVA, Bonferroni’s test, *P* < 0.001 and *P* < 0.05, respectively, Figure 9E,F, respectively, *n* = 4 in all cases). In addition, microglial activation was assessed by analyzing the expression of MHCII, a marker of activated microglia and macrophages (Butovsky et al., 2005). The quantity of Iba1-positive cells co-labeled by anti-MHCII antibody was higher in 12-month-old mice than in 1-month-old animals, but no significant differences were observed between wild-type and TLR4 KO mice in terms of the number of MHCII-positive cells (Figure 9E,F). These data indicate that TLR4 deficiency reduces the density of retinal microglia cells, without affecting the state of microglia activation, under normal conditions.

Conversely, the mean number of astrocytes in TLR4 KO mice was 95.2% of that observed in WT mice, without reaching signification (not shown). Müller cells morphology showed similar features in TLR4 KO and control mice (not shown).

## Discussion

Mammalian TLRs have relevant roles in embryogenesis, development of neurons and neural circuits, learning and memory, with regulatory roles on the processes of proliferation, differentiation, outgrowth, plasticity and neuron survival (Rolls et al., 2007; Shechter et al., 2008; Hanke and Kielian, 2011; Okun et al., 2011; Barak et al., 2014; Grasselli et al., 2018). Thus, it is expected that TLRs contribute to the development and maintenance of retinal structure and function. Our hypothesis is that TLR4 has a relevant role in the morphology and function of the retina under physiological conditions. TLR deficient mice have extensively proven to be useful to elucidate the role and relevance of TLRs. Thereby, we used a TLR4 KO mouse to assess the influence of TLR4 on postnatal retinal morphology and function.

Our results show that, in healthy conditions, TLR4 deletion alters both the structure and function of the retina in postnatal mice, and that this effect remains throughout life. We have demonstrated that TLR4 deletion reduces the mice retinal responsiveness from P20 to P360, with lower scotopic a- and b- waves, reduced oscillatory potentials and lower visual acuity. The significant reduction in retinal function can be explained, at least in part, by both the reduction in bipolar cells and the loss of their dendritic arbors in the OPL of TLR4 KO mice. We report a significant decrease in the density of bipolar cells at P20, P30, and P360, with a consequent reduction in their dendritic arborization. Our results agree with those of previous studies showing that LPS, acting through TLR4 in astrocytes, can induce changes in the dendritic pattern of hippocampal neurons (Shen et al., 2016). More recent studies have also found that TLR4 regulate GABAergic synapse function under pathological situations in psychologically stressed animals (Varodayan et al., 2018). Shechter and collaborators showed that retinal progenitor cell proliferation is enhanced in TLR4-deficient mice during the early postnatal period (Shechter et al., 2008) in ciliary epithelium and *ora serrata*. As the number of neuronal cells is not increased in adult retinas, we hypothesize that the known process of programmed elimination of extra neurons in the CNS could be responsible for that (Harris and McCaig, 1984; Burke, 2003; Kim and Sun, 2011). Despite the discrepancy between the decrease in density and dendritic arborization of bipolar cells and the lack of change in photoreceptor numbers and morphology, images of transmission electron microscopy did not show differences in the structure of the photoreceptor spherules.

Recent data indicate that many molecules associated with the immune system regulate circuit development and plasticity in healthy brains (Wu et al., 2015). Microglial cells, the CNS resident immune phagocytes, play an essential role in the formation and maintenance of synaptic networks under physiological conditions (Tremblay, 2011; Salter and Beggs, 2014; Mallard et al., 2018; Sominsky et al., 2018). Accordingly, TNF-alpha and different cytokines secreted by fetal microglia have a crucial role in CNS development, and synapse formation, refinement and function. As the brain develops, neurons form an overabundance of synaptic connections, many of which are later removed during what is known as synapse pruning, a process crucial to proper brain connectivity (Deverman and Patterson, 2009; Schafer et al., 2013; Wu et al., 2015). Moreover, microglia engulf synaptic material in an active manner and play an important role in postnatal synaptic pruning in mice (Paolicelli et al., 2011; Schafer et al., 2012, 2013). In line with this, disrupting microglia-specific signaling produced sustained deficits in synaptic connectivity (Schafer et al., 2012, 2013; Zhan et al., 2014; Pagani et al., 2015). Also, a recent work by Jobling et al. has shown that in postnatal neural development, the absence of microglial Cx3CR1 signaling induced retinal disfunction and photoreceptor loss (Jobling et al., 2018). Among the TLRs, TLR1 to TLR9 are expressed in microglia (Parkhurst and Gan, 2010), and microglial activation depends on TLRs (Olson and Miller, 2004; Lehnardt, 2010), including TLR4 (Fellner et al., 2013; Yao et al., 2013). Moreover, previous works have shown that TLR4 play a part in the activation of retinal microglial cells (Kohno et al., 2013; Lee et al., 2015). Our data indicate that TLR4 deficiency reduces microglial density in the retina of healthy mice. This is consistent with previous data showing that microglial phagocytosis of degenerating axons is reduced in TLR4 KO mice (Rajbhandari et al., 2014). Given the key role of microglia in developing and maintaining the structure and function of the CNS, the retinal microglia deficiency could be responsible, at least in part, for the observed changes in the structure and function of mouse retinal neurons. In this sense, Kashima and Grueter have recently shown that TLR4 influences NMDA-receptor synaptic transmission and plasticity, likely through TLR4 expressed in microglial cells (Kashima and Grueter, 2017).

Other works have previously demonstrated a role for TLRs in the development of neuronal circuits (Okun et al., 2011). In *Drosophila*, Rose and collaborators have shown that Toll receptors are involved in the synaptic initiation of motoneurons and reported the presence of anomalous innervations in Toll mutants (Rose et al., 1997), which can alter synaptic target recognition (Ritzenthaler and Chiba, 2003). Also, TLRs activation can induce synaptic dysfunction in a pathological situation (Salminen et al., 2008). The activation of TLRs in an inflammatory process triggers the production of several cytokines, as IL-1beta or IFN-gamma that can regulate synaptic plasticity. In this sense, in the case of Alzheimer’s disease, it has been shown that the IL-1beta secreted by microglia cells decreases the synthesis of synaptophysin (Li et al., 2003) and that IFN-gamma can decrease the rate of synapse formation, as shown in rat-cultured sympathetic and hippocampal neurons (Kim et al., 2002).

Nowadays it is accepted that TLRs can have both, beneficial and harmful effects on CNS development and neuroplasticity, which can vary depending on the physiological or pathological state and tissue homeostasis (Hanke and Kielian, 2011; Okun et al., 2011). In a balanced scenario, an acute TLR activation may tip the balance toward tissue damage or pathology, while a chronic or limited response might be necessary for maintaining the homeostatic balance (Hanke and Kielian, 2011).

Our observation that bipolar cell numbers and their dendritic arbors are reduced in TLR4 KO adult mice also suggests that TLR4 is required for the development of a mouse retina with the correct structure and function. Whether this mechanism acts mainly by altering cellular proliferation or differentiation processes during embryogenesis, through errors in synapses formation or both, remains to be elucidated. Although more studies are needed to clearly identify their function, altogether these results indicate that TLR4 may be involved in establishing the correct cellular structure of the retina during embryogenesis.

Toll-like receptors are potential therapeutic targets and there is increasing hope that TLRs agonists and antagonists could be used in the treatment of CNS disorders. Since TLR4 activation is related to the progression of several neurodegenerative disorders, mainly by triggering an inflammatory process through the secretion of proinflammatory cytokines by the microglial cells, the use of TLR4 agonists and antagonists could balance or counteract the progression of the diseases or the therapeutic approach. Beyond its role as an immune system receptor, a complete understanding of the TLR signaling pathways is needed to understand the molecular bases of retinal development and/or degeneration, and also to prevent the effects on CNS balance of the use of TLRs agonists and antagonists to treat immune and non-immune pathologies.

Overall, our study suggests that genetic deletion of toll-like receptor 4 causes functional alterations in terms of visual response and acuity, probably through the loss of bipolar cells and microglia. However, the expression of TLR4 does not appear to be essential for the processing of visual information in the retina. Identification of the role of TLR4 on the structure and function of the adult retina has implications for research into the involvement of TLRs in physiological and pathological conditions, and may contribute to the development of new therapeutic options for both infectious and non-infectious diseases.

## Author Contributions

AN, OK, IO-L, LC, EdJ, and VG-V collected and analyzed the data and revised the manuscript. VM designed the experiments and drafted the manuscript. NC designed the study with the assistance of VM and PL, provided study material, and revised the manuscript. PL designed the experiments, collected and analyzed the data, and revised the manuscript. All authors have read and approved the final submitted manuscript.

## Conflict of Interest Statement

The authors declare that the research was conducted in the absence of any commercial or financial relationships that could be construed as a potential conflict of interest.

## Footnotes

1 http://rsbweb.nih.gov/ij/index.html

## Abbreviations

DAMP: damage-associated molecular pattern
ERG: electroretinography
Iba1: ionized calcium-binding adaptor molecule 1
INL: inner nuclear layer
IPL: inner plexiform layer
LPS: lipopolysaccharide
ONL: outer nuclear layer
OPL: outer plexiform layer
PAMP: pathogen-associated molecular pattern
PB: phosphate buffer
PKC: protein kinase C
SEM: standard error of the mean
TEM: transmission electron microscopy
TLR: Toll-like receptor
